# A Reactive ^1^O_2_ - Responsive Combined Treatment System of Photodynamic and Chemotherapy for Cancer

**DOI:** 10.1038/srep29911

**Published:** 2016-07-22

**Authors:** Xiaojun Wang, Guoqing Meng, Song Zhang, Xinli Liu

**Affiliations:** 1 Tianjin Institute of Industrial Biotechnology, Chinese Academy of Sciences, Tianjin 300308, China; 2 University of Chinese Academy of Sciences, Beijing 100049, China; 3 Life Sciences Department, Heze University, Heze 274015, China; 4 Shandong Provincial Key Laboratory of Microbial Engineering, Qilu University of Technology, Jinan 250100, China

## Abstract

The development of reactive oxygen species (ROS)-responsive drug delivery and drug release has gradually attracted much attention in recent years as a promising therapeutic strategy. Singlet oxygen (^1^O_2_) as the major ROS species is widely used in photodynamic therapy (PDT) of cancer. In the present study, we introduce a combined treatment using ROS-sensitive thioketal (TK) linkage as a linker between upconversion nanoparticles (UNs)-based PDT and doxorubicin (DOX)-based chemotherapy. UNs can not only play a role in PDT, but can also be used as a nanocarrier for drug delivery of DOX. Moreover, the products of ^1^O_2_ during PDT are able to cleave TK linker inducing the release of DOX which can further achieve the goal of chemotherapy. By using this ^1^O_2_-responsive nanocarrier delivery system, DOX can easily reach the tumor site and be accumulated in the nuclei to effectively kill the cancer cells, and therefore decreasing the side effects of chemotherapy on the body. Thus, PDT also has the function of controlling drug release in this combination treatment strategy. Compared with monotherapy, the combination of PDT with chemotherapy also possesses excellent drug loading capability and anticancer efficiency.

UNs-based PDT has recently attracted tremendous attention for their applications in the field of cancer treatment owing to their unique photomechanical properties. Under the excitation of 980 nm near-infrared (NIR) light, ^1^O_2_ can be generated via resonance energy transfer from UNs to photosensitizer MC540 and cause cellular damage and subsequent cell death. The use of NIR light with the range of wavelengths from 650 to 1350 nm can afford its maximum depth of penetration in tissue, and exhibit an ideal PDT efficiency^1^,^2^,^3^,^4^. A number of studies have shown that the combining PDT with chemotherapy could overcome drug resistance by invoking multiple anticancer mechanisms^5^,^6^,^7^.

DOX is one of the most widely used chemotherapeutic agents in the treatment of a wide range of cancers and is combined with UNs-based PDT in this work. DOX intercalates DNA and inhibits the progression of the enzyme topoisomerase II, thereby inhibits cell growth and reproduction by interferencing DNA replication^8^,^9^,^10^,^11^,^12^. UN/MC540 also acted as a nano-sized drug carrier for DOX with high drug concentrations to the targeted cancer cell and reduced toxicity to normal tissue^13^,^14^,^15^,^16^,^17^.

How to made DOX release and passed through the nuclear pore complex to cause DNA damage was the key of this combined treatment. The thioketal group, which could be readily cleaved by ^1^O_2_, was used as the linker between UNs/MC540 and DOX. ^1^O_2_, generated by PDT under the excitation of NIR light, was able to trigger the release of doxorubicin from the surface of UN/MC540 in cancer cells^18^,^19^,^20^. And then DOX molecules more easily reached cell nucleus and led to DNA damage efficiently. Meanwhile, in addition to control drug release, ^1^O_2_ could also cause cancer cell damage to compensate for the detrimental effects of chemotherapy^21^,^22^,^23^. Furthermore, UN/MC540-DOX could be modified by folic acid (FA) to enhance the binding affinity of nanoparticles toward cancer cells^11^,^24^,^25^,^26^. In this work, we developed FA-UN/MC540-DOX as a combination agent for *in vivo* PDT treatment of tumors (Fig. 1).

## Results

### Preparation and Characterization of combination nanoparticles

**Figure 2:**
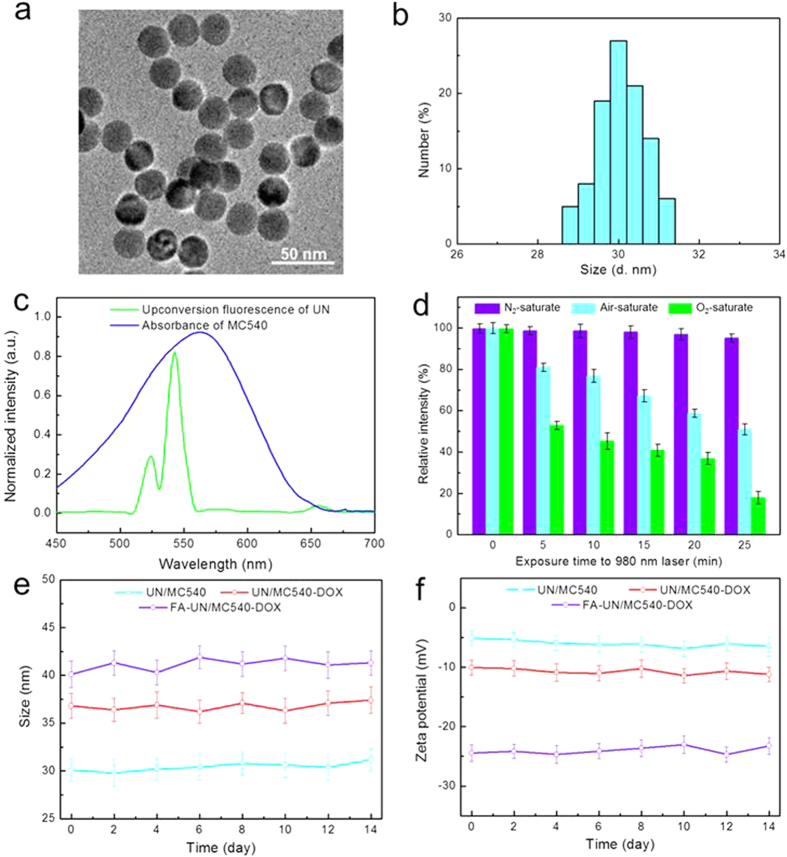
(**a**) TEM image of as-synthesized UN/MC540. (**b**) DLS analysis of size distribution variation of UN/MC540. (**c**) The fluorescence emission spectrum of UNs under 980 nm laser excitation and the absorption spectra of MC540. (**d**) Oxygen-dependent fluorescence decay of ABDA at 400 nm. (**e**,**f**) presentation variation of size and zeta potential of nanoparticles in cell culture within two weeks.

NaYF4:Yb/Er UNs were synthesized using a modified method reported by literature^27^,^28^,^29^. The obtained hydrophobic NaYF4:Yb,Er/NaYF4 core/shell upconversion nanoparticles were converted to hydrophilic ones using an amphiphilic block copolymer, DSPE-PEG (1,2-distearoyl-sn-glycero-3-phosphoethanolamine-polyethylene glycol). The as-synthesized UNs were highly soluble in water^30^,^31^,^32^,^33^,^34^. Figure 2a showed a TEM (transmission electron microscopy) image of the hydrophilic UNs and presented uniform structure with an average size of around 25 nm. DLS (dynamic light scattering) analysis exhibited UN/MC540 with a hydrated particle size of 30 nm (Fig. 2b). UN/MC540-DOX and FA-UN/MC540-DOX were prepared by adding a certain percentage of MC540, DSPE-PEG-DOX and DSPE-PEG-FA in the process of hydrophilic conversion. Their particle size and zeta potential were well maintained in cell culture media by DLS measurements during two weeks (Fig. 2e,f). The slightly increased particle size and zeta potential indicated the success of the FA anchoring on the membrane surface by comparing these three nanoparticles (Table 1). Based on the HPLC analysis, the DOX and FA contents in conjugate DSPE-PEG-DOX and DSPE-PEG-FA were calculated to be 16.4 and 13.2 wt%, respectively. And the loading percentage DOX in FA-UN/MC540-DOX was calculated to be 7.2%.

### Ability to produce singlet oxygen in solution

Under 980 nm laser excitation with proper intensity, the emission band at 540 nm was the main emission of upconversion visible fluorescence from UNs, which matched well with the absorption of photosensitizer MC540 (Fig. 2c). The character of MC540 molecular absorbed on the surface of UN allowed for fluorescence transfer from the UN to MC540, thereby the activating MC540 could generate cytotoxic ^1^O_2_ with surrounding oxygen molecule. We measured the production of ^1^O_2_ with light in three different concentration systems of oxygen gas, including N_2_, Air and O_2_-saturate aqueous solution containing the dye 9,10-anthracenediyl-bis(methylene)dimalonic acid (ABDA). ABDA was used as a water-soluble ^1^O_2_ monitor by its fluorescence decay due to the reaction with ^1^O_2_^35^,^36^,^37^. These results provided evidence that UN/MC540 could generate ^1^O_2_ using oxygen molecular existed in surrounding environment. In addition, the yield of ^1^O_2_ was affected by illumination time at the same concentration (Fig. 2d).

### Detection of ^1^O_2_ in solution and cancer cells

**Figure 3:**
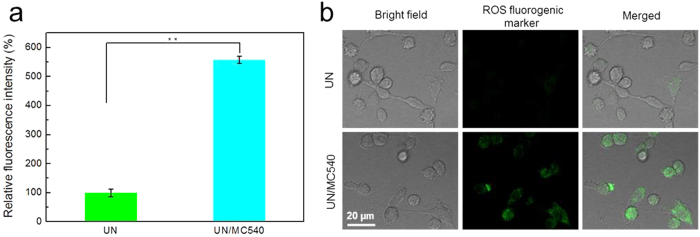
The generated singlet oxygen of PDT in solution (**a**) and B16 cells (**b**).

Singlet oxygen in cancer cells was detected using ROS Kit (Reactive Oxygen Species Assay Kit) by flow cytometry and confocal laser scanning microscopy (CLSM) methods under 980nm laser irradiation (0.5 W/cm^2^)^38^. As compared to the faint fluorescence signal in the UN group, significant amounts of ^1^O_2_ were produced in UN/MC540 group. These results further demonstrated that UN/MC540 possessed the ability to generate ^1^O_2_ efficiently and could be used in PDT for cancer cells (Fig. 3a,b).

### ROS-responsive drug release

**Figure 4:**
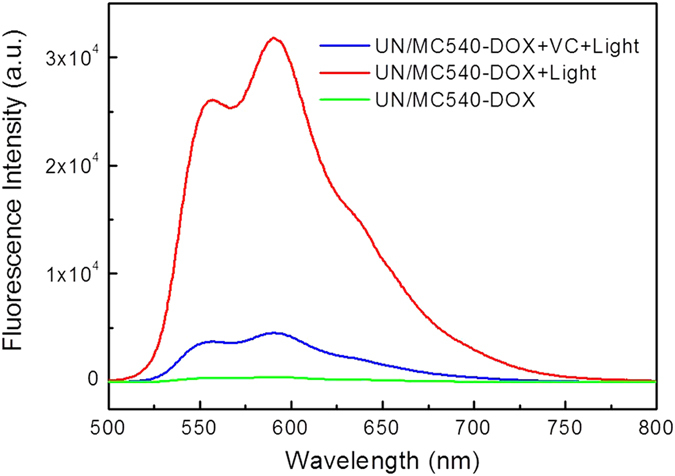
Detection of the ^1^O_2_-triggered drug release of DOX.

In addition to kill cancer cells, the generated ^1^O_2_ in PDT could also control the drug release since DOX was covalently conjugated to UN/MC540 by the ^1^O_2_-responsive TK linker^39^,^40^. To certify the ability of ^1^O_2_-responsive drug release, aqueous solution of UN/MC540-DOX was treated by 5 min illumination with power density of 0.5 W/cm^2^. After that, nanoparticles were removed by centrifugation (20,000 rpm, 5 min), and supernatant was collected in another tube to measure DOX fluorescence intensity (488 nm excitation and 585 nm emission). The concentration of DOX released from UN/MC540 was determined by its fluorescent intensity. Compared with the untreated group, the light promoted DOX release from nanoparticle. To further demonstrate this was a ^1^O_2_-induced DOX release mechanism, vitamin C (VC) as a ROS scavenger was added into the light treated group. The fluorescence from the DOX was significantly inhibited, thus further confirming the generated ^1^O_2_ under 980 nm laser excitation acted on the TK linker and induced DOX release from nanoparticle (Fig. 4).

### Improved tumor cell uptake by FA

**Figure 5:**
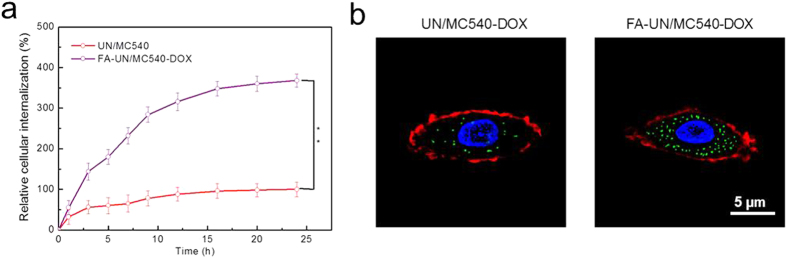
Targeting ability evaluation by cell uptake of nanoparticles using flow cytometry (**a**) and CLSM methods (**b**).

To achieve efficient accumulation in cancer cells, FA conjugated on the surface of FA-UN/MC540-DOX was used as cancer cell targeting molecule. FA was able to specifically bind to FA receptors over-expressed in a range of tumor cells with a high affinity, enabling FA-UN/MC540-DOX deliver efficiently to cancer cells without causing harm to normal cells^24^,^41^,^42^,^43^. We therefore evaluated the FA effect on cancer cell uptake. As shown in Fig. 5a, FA-UN/MC540-DOX displayed the higher intracellular concentration than UN/MC540-DOX because of the FA modification on the nanoparticle. Similar results were obtained by CLSM imaging. As seen in Fig. 5b, cell nucleus (blue) was stained with Hoechst, cell membrane (red) was labeled with CellMask™ Deep Red Plasma membrane Stain, and FA-UN/MC540-DOX (green) was detected at 980 nm and corresponding fluorescent images at 540 nm were taken by CLSM^44^. These results together demonstrated that the cellular uptake of FA-UN/MC540-DOX was significantly promoted with the assistance of FA. Nanoparticles internalization played a pivotal role for PDT and chemotherapy.

### Combination therapy evaluation by cytotoxicity

**Figure 6:**
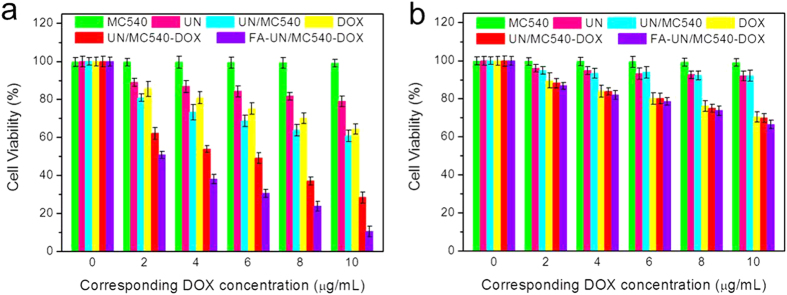
Efficacy of PDT treatment with (**a**) or without (**b**) light were assessed by measuring cell viability.

To test combination treatment effect, we assessed the cytotoxicity of MC540, UN, UN/MC540, free DOX, UN/MC540-DOX, and FA-UN/MC540-DOX with light using CCK-8 (cell counting kit-8) assay. As shown in Fig. 6a, MC540 and UN exhibited little cytotoxicity at the every corresponding theoretical DOX concentration. On the contrary, both UN/MC540 and free DOX showed severe cytotoxicity to B16 cells due to the PDT and traditional chemotherapy, respectively. As expected, UN/MC540-DOX and FA-UN/MC540-DOX exhibited excellent anti-tumor activities by combining these two methods of treatment for cancer cells. Notably, FA-UN/MC540-DOX showed a significant improvement in antitumor therapeutic efficacy because of FA targeted modification. The corresponding IC50 (concentration resulting in a 50% inhibition of cell growth) values for free DOX and UN/MC540 were more than 10 μg/mL, while that of FA-UN/MC540-DOX was only about 2 μg/mL. However, in the case of no light treatment, UN/MC540-DOX and free DOX displayed similar effects of inhibition of cancer cells. UN/MC540 behaved little cytotoxicity to cells in every concentration. Without light the tumor cytotoxic of nanoparticle was mainly from chemotherapeutic agents DOX (Fig. 6b). These results demonstrated the enhanced antitumor activity of FA-UN/MC540-DOX compared with PDT or chemotherapy alone^45^.

### *In vivo* anticancer efficient

**Figure 7:**
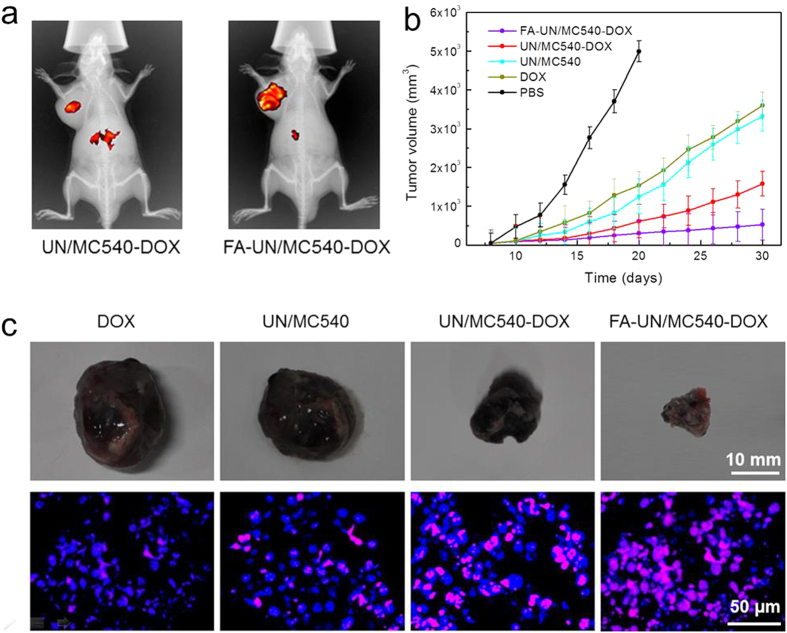
(**a**) Targeting ability test *in vivo* combination treatment of a subcutaneous tumor model injected with UN/MC540-DOX and FA-UN/MC540-DOX. (**b**) Suppression of B16 tumor growth in mice by FA-UN/MC540-DOX compared with other agents groups. (**c**) Tumor images and the corresponding nuclear apoptosis analysis by TUNEL staining.

DSPE-PEG endowed nanoparticles with stealth behavior to protect them from the RES (reticulo-endothelial system) and easily reached the tumor tissue via EPR effect. Besides, the grafting FA molecules onto the nanoparticles refered to active targeting and aimed to increase specific cancer cells uptake at the tumor site by FA receptor-mediated endocytosis^32^,^46^,^47^,^48^,^49^. The injection of Cy5.5-labeled FA-UN/MC540-DOX was more likely to accumulate at the tumor site than non-target nanoparticle in the process of PEG-induced long blood circulation (Fig. 7a). Having demonstrated the tumor targeting ability of FA, we moved on to explore the antitumor efficacy of different nanoparticles. In comparison with PBS group, all treatment groups showed varying degree of tumor suppression within three weeks. Among them, combination treatment groups exhibited better antitumor effect than free DOX and UN/MC540 group. Notably, targeted modification group showed the greatest anti-cancer effect. Likewise, nuclear apoptosis of cancer cells analysis by TUNEL staining was in agreement with the above results. FA-UN/MC540-DOX induced the greatest cell apoptosis and the tumor was also the smallest, again confirming the success of our combination therapy strategies for cancer cells (Fig. 7b,c).

## Discussion

In conclusion, we have developed an efficient combination control approach for tumor therapy, which consisted of UN-based PDT and chemotherapy. FA-UN/MC540-DOX was applied to an ideal nanoscale drug delivery device with high drug loading for DOX. And the molar percentage of PEG introduced onto the surface of UN/MC540 endowed the nanocarrier with superior stealth ability to enhance circulation time, and led to tumor accumulation of DOX via EPR effect. Moreover, FR-targeted modification nanoparticle increased cellular uptake and promoted more efficient DOX delivery *in vivo*. Once FA-UN/MC540-DOX arrived at the tumor site or was uptaken by cancer cells, the produced ^1^O_2_ in the PDT could quickly trigger the release of DOX from this nanoparticle via cleavage of TK linker. Meanwhile, free DOX was easily able to travel through the nuclear envelope to kill cancer cells efficiently. In addition, UN/MC540-based PDT could also overcome the limitations found in conventional chemotherapy. Thus, our present combination treatment with controlled-release ability exhibits a better anticancer efficacy and paves the way for further research on the clinical application.

## Methods

### Reagents and materials

LnCl_3_·xH_2_O (Ln = Y, Yb, Er; x ≈ 5, 99.9%), ammonium fluoride (NH_4_F, 96%), 1-octadecylen (ODE) and oleic acid (OA) were purchased from Alfa Aesar Reagent Company. N-hydroxysulfosuccinimide (NHS), 1-ethyl-3-(3-dimethylamino-propyl) carbodiimide (EDC), 9, 10-Anthracenediyl-bis(methylene)dimalonic acid (ABDA), vitamin C, merocyanine 540 (MC540) and DOX were obtained from Sigma Aldrich (USA). DSPE-PEG_2000_ (1, 2-distearoyl-sn-glycero-3-phosphoethanolamine, DSPE), DSPE-PEG_2000_-NH_2_ and DSPE-PEG_2000_-Folate were purchased from Avanti Polar Lipids (USA). All other reagents were of analytic grade.

Roswell Park Memorial Institute (RPMI) 1640 Medium, Hoechst, Cy5.5 dye, and CellMask™ Deep Red Plasma membrane Stain were purchased from Life technologies, and Mitochondrion-Selective Probe were supplied by Invitrogen (USA). Cell Counting Kit-8 (CCK-8) was bought from Beyotime (China). B16 cells and BALB/C mice were obtained from the Peaking University Health Science Center (China). All animal experiments were performed in accordance with the principles of care and use of laboratory animals and were approved by the experiment animal administrative committee of Peaking University Health Science Center.

### Preparation of UN/MC540-DOX and FA-UN/MC540-DOX

NaYF_4_:Yb,Er (18, 2 mol %) UNs were synthesized as follows: a mixture of YCl_3_ (0.8 mmol), YbCl_3_ (0.8 mmol) and ErCl_3_ (0.02 mmol) dissolved in 2 mL of methanol was mixed in a 50 mL flask. Oleic acid (6 mL) and 1-octadecene (ODE) (15 mL) were added to the above solution, and the mixture was stirred at room temperature for 10 minutes. Methanol solution (5 mL) containing NaOH (2.5 mmol) and NH_4_F (4 mmol) were added into this flask. After that, methanol was evaporated by heating at 100 °C, and the mixture was maintained at 300 °C for 1 h under argon protection. After cooling the reaction mixture to room temperature, medium solution (10 mL) was collected as the core for the later fabrication of core/shell NaYF_4_:Yb,Er/NaYF_4_ UNs. YCl_3_ (1 mmol), NaOH (2.5 mmol) and NH_4_F (4 mmol) dissolved in methanol (5 mL) were added into the collected core solution to prepare core/shell UNs. The solution was stirred for 30 min and heated to remove methanol. After being degassed and maintained under argon protection, core/shell UNs were obtained by centrifugation and washed for three times^27^.

**Figure 8:**
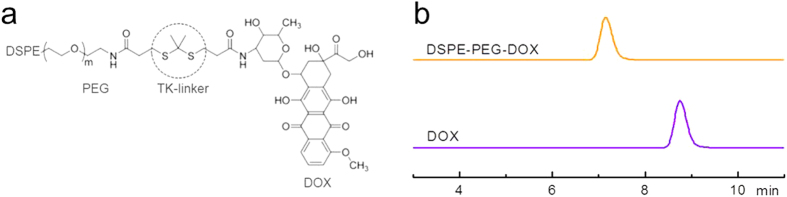
(**a**) Chemical structure of the PEG-TK-DOX. (**b**) HPLC chromatograms of DOX and DSPE-PEG–DOX. Retention times were about 8.7 min, and 7.1 min for DOX and DSPE-PEG-DOX conjugate, respectively.

TK and DSPE-PEG-DOX were synthesized according to the method reported previously (Fig. 8a)^18^,^20^. DOX showed the retention at 8.7 min and DSPE-PEG-DOX showed retention at about 7.1 min with major single peak (Fig. 8b). To prepare UN/MC540-DOX, 10 mg of DSPE-PEG-DOX and 5 mg of MC540 were added into a 25 mL flask containing 10 mg of UNs and 5 mL of CHCl_3_. This mixture was stirred overnight at room temperature. In this reaction, 5 mg of DSPE-PEG_2000_-Folate was added to prepare targeted modification of PEG-TK-DOX nanoparticles.

### Loading percentage of MC540 and DOX in UNs

MC540 and DOX encapsulated within the UNs were determined in triplicate by HPLC with UV detection at 227 nm and 290 nm (LC-20AT, Shimadzu), respectively. The loading percentage was calculated according to the following formula:


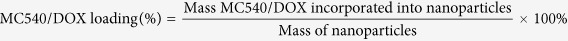


### Detection of ^1^O_2_ production in solution

Irradiation of UNs with a 980 nm laser resulted in emission of visible light at 540 nm peak which matched well with the absorption of the MC540. ^1^O_2_ was generated via energy transfer from UNs to MC540 upon 980 nm NIR excitation. We speculated that the presence or content of oxygen molecules would affect the fluorescence spectrum at 540 nm. The generation stable peroxide because of the water soluble ABDA reaction with ^1^O_2_ can lead to the falling absorption peak of ABDA at 400 nm. Thus, ABDA was used as a ^1^O_2_ trapping reagent in aqueous solution. So we examined the production of ^1^O_2_ in three different conditions, including the N_2_-saturated, O_2_-saturated, and air-saturated aqueous solution. In a typical experiment, 1 mL of aqueous solution containing 5 μM of ABDA dye and 5 μg/mL UN/MC540 was placed in these three aqueous solutions with different illumination time.

### ^1^O_2_ detection in live cells

Reactive Oxygen Species Assay Kit was used to detect the generation of ^1^O_2_ which resulted from the photosensitization experiments in B16 cells. The nanoparticles-loaded cells were treated with the culture medium containing the DCFH-DA solutions (10 μmol/L) for 20 min at 37 °C. After that removed the mixture and washed cells with serum-free culture three times. The following cells were subjected to photosensitization experiment by 980 nm NIR laser irradiation for 2 min. And then fluorescent images of cells were promptly captured by excitation at 488 nm and emission at 525 nm using confocal laser scanning microscopy (CLSM, TCS SP5, Leica).

### FA-mediated cellular uptake test

B16 cells were routinely cultured in RMPI 1640 medium with 10% FBS in a humidified incubator with 5% CO_2_ at 37 °C. After 24 h, cells were incubated with UN/MC540-DOX and FA-UN/MC540-DOX (10 μg/mL) at 37 °C for different time intervals. Cells were rinsed with PBS (pH 7.2) solution softly and detached by 0.2% trypsin. And removed typsin by centrifugation at 1000 rpm for 5 min. Cells were then harvested and re-suspended in 100 mL PBS and examined by flow cytometry using a CyAn ADP 9 color flow cytometer (Beckman Coulter). Using the same method, B16 cells were grown in a culture dish at a density of 1 × 10^6^ cells/dish and incubated at 37 °C for 24 h. Cells in dishes were incubated with culture containing 200 μl of the above two kinds of nanoparticles. Then removed medium and washed cells with PBS (pH 7.2). The cell nucleus and cell membrane were stained with Hoechst and CellMask™ Deep Red Plasma membrane Stain, respectively. FA-UN/MC540-DOX was detected at 980 nm and corresponding fluorescent images at 540 nm was taken by CLSM (TCS SP5, Leica).

### Cytotoxicity evaluation

Cytotoxicity of UN nanoparticle was evaluated by CCK-8 kit assay. B16 cells were seeded in 96-well plates at the density of 5 × 10^5^ cells/well and cultured for 24 h at 37 °C. Original medium in each well was refreshed with 200 μL medium containing serial dilutions of UNs (corresponding DOX concentration was 0–10 μg/mL). After incubation for 48 h, 20 μL of CCK-8 kit was added to all wells, and then cells were continued to be incubated at 37 °C with 5% CO_2_ for 4 h. Infinite M200 microplate spectrophotometer (Tecan, Mannedorf, Switzerland) was used to detect the absorbance at 540 nm. Percent viability was normalized to cell viability in the absence of the samples.

### *In vivo* anticancer efficient

To demonstrate the targeting ability of FA-UN/MC540-DOX *in vivo*, Cy5.5 dye was loaded into this nanoparticle according to the method described in the preparation of MC540-loaded nanoparticles. B16-bearing mice were injected intravenously with 5 μg of FA-UN/MC540-DOX and UN/MC540-DOX in 100 μL of PBS (pH 7.2), respectively. Eight hours after the intravenous administration, mice were anesthetized and scanned using *in-vivo* imaging system with an excitation at 670 nm and an emission filter at 700 nm.

In the subcutaneous B16 melanoma model, treatments were started when the average tumor volume reached about 100 mm^3^, and mice were randomly divided into five groups. Five groups of eight mice each were intravenously administered PBS (100 μL, pH 7.2), DOX, UN/MC540, UN/MC540-DOX and FA-UN/MC540-DOX (1 mg/kg of DOX) once every two days, and then tumor sites were irradiated with a 980 nm laser. Tumor sizes were measured with a digital caliper every other day. And tumor volume was calculated by the formula (LxW^2^)/2, where L was the long and W was the short tumor diameter (mm). Until tumor volume reached 5000 mm^3^ in PBS group, mice were sacrificed for humane reasons. In other groups, one mouse of each group was sacrificed and tumor tissue was removed for apoptotic analysis. Detection of apoptotic cells by TUNEL (terminal transferase-mediated dUTP nick end labeling) staining accorded to the protocol from Thermo Fisher Scientific.

## Additional Information

**How to cite this article**: Wang, X. *et al*. A Reactive ^1^O_2_ - Responsive Combined Treatment System of Photodynamic and Chemotherapy for Cancer. *Sci. Rep.* **6**, 29911; doi: 10.1038/srep29911 (2016).

## Figures and Tables

**Figure 1:**
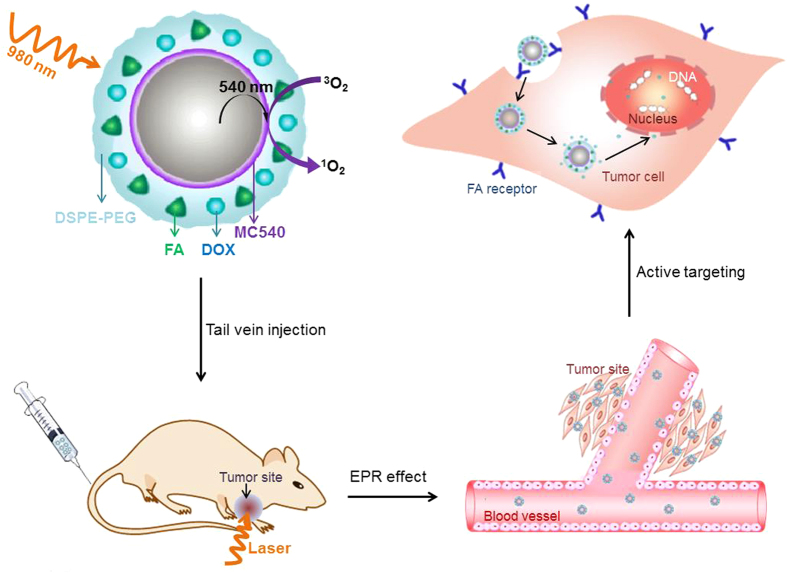
Schematic illustration of UNs-based photodynamic and chemotherapy for cancer; the long blood-circulation time of nanoparticles enabling their accumulation at the tumor site by enhanced permeability and retention (EPR) effect; and active targeted feature of the agents facilitating the uptake of the cancer cell.

**Table 1:** Particle size and zeta potential of different PDT agents.

Groups	Particle size (nm)	Zeta potential (mV)
UN/MC540	30 ± 1.2	−5.34 ± 1.1
UN/MC540-DOX	36 ± 1.4	−10.22 ± 1.4
FA-UN/MC540-DOX	40 ± 1.5	−24.65 ± 1.5
